# Dataset containing spectral data from hyperspectral imaging and sugar content measurements of grapes berries in various maturity stage

**DOI:** 10.1016/j.dib.2022.108822

**Published:** 2022-12-12

**Authors:** Maxime Ryckewaert, Daphné Héran, Carole Feilhes, Fanny Prezman, Eric Serrano, Aldrig Courand, Silvia Mas-Garcia, Maxime Metz, Ryad Bendoula

**Affiliations:** ITAP, Univ Montpellier, INRAE, Institut Agro, Montpellier, IFV, 1920 Route de Lisle-sur-Tarn, 81310 Peyrole, France

**Keywords:** Hyperspectral imaging, Digital agriculture, Chemometrics, Grape berries, Sugar content, Spectroscopy

## Abstract

In the dataset presented in this article, two hundred and seventy four trays containing one hundred berries were measured by a hyperspectral camera in the visible/near-infrared spectral domain. This dataset was formed to study the use of hyperspectral imaging for maturity monitoring of grape berries [2]. This dataset contains reflectance spectra from hyperspectral camera of grape berries of three different varieties and chemical composition (sugar content).

**Specifications Table**

**Table:** 

Subject	Analytical Chemistry: Spectroscopy
Specific subject area	Maturity monitoring of grape berries with sugar content and spectral measurements
Type of data	Table
How the data were acquired	Reflectance spectra were acquired with a hyperspectral camera (Specim IQ, Specim, Finland) having a spectral range from 400 nm to 1000 nm and a spectral resolution equal to 7 nm. Sugar content measurements were performed on berry musts with a refractometer (HI-96816, Hanna Instruments).
Data format	Raw
Description of data collection	Three different grape varieties were collected in Gaillac (France), in summer 2020. Grape berries were then sorted using NaCl densimetric baths and were placed on a tray for hyperspectral acquisition. Reflectance spectra were obtained with pixel selection corresponding to berries. Sugar content were measured on berry musts with a refractometer (HI-96816, Hanna Instruments).
Data source location	*• Institution: Institut Français de la Vigne et du vin (IFV)* *• City/Town/Region: Peyrole, Tarn, Occitanie* *• Country: France*
*Data accessibility*	*Repository name: Mendeley Data* *Data identification number: gjwx64sgkp.1* *Direct URL to data:*https://doi.org/10.17632/gjwx64sgkp.1
*Related research article*	*For a published article: [*2*]* *Aldrig Courand, Maxime Metz, Daphné Héran, Carole Feilhes, Fanny Prezman, Eric Serrano, Ryad Bendoula, and Maxime Ryckewaert. Evaluation of a robust regression method (RoBoost-PLSR) to predict biochemical variables for agronomic applications: Case study of grape berry maturity monitoring. Chemometrics and Intelligent Laboratory Systems, 221:104485, February 2022. ISSN 01697439. doi:*https://doi.org/10.1016/j.chemolab.2021.104485

**Value of the Data**

• These spectra were acquired under controlled conditions over a range of sugar values representative of all stages of maturity.
• This dataset is useful for testing and comparing prediction methods.
• This dataset can be used to build models to predict the sugar content of grapes.
• This dataset is intended for scientists to test new methods (variable selection, data exploration, regression methods) or to have reference data to guide their future experiments.

## 1 Data Description

Chemical and Nir spectra measurements were made on 274 samples (one sample is composed by one hundred berries) from three different grape varieties (Table 1). The table containing the dataset (**DATASET.csv**) is represented so that the rows correspond to the samples and the columns correspond to the variables. The first column corresponds to the tray key, the second column to the variety, the third column to the sugar content and the following columns correspond to reflectance spectra values obtained on the indicated spectral bands. For the three varieties, sugar content values are similar and comprised between 100 and 300 (g/L), representative of all stages of maturity.

**Table 1:** Number of observations (hyperspectral images and sugar contents) constituting the whole data set for the three grape varieties, Syrah, Fer and Mauzac.

	Syrah	Fer	Mauzac
Number of observations	126	63	85

Grape berries were sorted using NaCl densimetric baths and were placed on a tray for hyperspectral acquisition.

From these one hundred berries, an average spectrum was calculated from each image. At this end, 274 reflectance spectra were obtained on three different grape varieties with two red grape varieties (Syrah and Fer Servadou) and one white grape variety (Mauzac).

To illustrate, PLSR results are shown for total sugar content for each grape variety (cf. Table 2).

**Table 2:** Cross-validation (cv) results of PLSR prediction models for total sugar content (latent variable number (LV), root mean square error (RMSE*_cv_*) and determination coefficient (R^2^*_cv_*))

Variety	LV	RMSE*_cv_* (g/L)	R2*_cv_*
Syrah	6	9.31	0.937
Fer Servadou	7	19.45	0.623
Mauzac	5	28.78	0.298

## 2 Experimental Design, Materials and Methods

### 2.1 Samples and analyses

The sampling of grape berries started one or two weeks after veraison and before harvest in summer 2020 on plots of the experimental vineyard Domaine Exp´erimental Viticole Tarnais located in Gaillac (France). The berries belonged to three grape varieties, two red grape varieties (Syrah and Fer Servadou) and one white grape variety (Mauzac). For each variety, thirty bunches were collected approximately once a week.

Grape berries were prepared in the laboratory. They were cut at the pedicel to preserve the whole fruit. Sorting was carried out by batches of the same degree of ripeness using sodium chloride (NaCl) baths. For this purpose, twelve NaCl baths of increasing concentrations from 70 to 190 g/L were prepared to classify the berries according to their berry density corresponding to sugar concentrations from 110 to 279 g/L. [1]. Berry musts were obtained with one hundred berries of the same degree of ripeness and sugar content measurements were made with a refractometer (HI-96816, Hanna Instruments).

### 2.2 Hyperspectral images and NIR spectra acquisition

Reflectance spectra were acquired before preparing a hundred berry must. These berries were placed on a tray to make the berries visible. These measurements were made with a hyperspectral camera (Specim IQ, Specim, Finland) with a spectral range of 400 nm to 1000 nm and a spectral resolution equal to 7 nm.

The camera was positioned at 1.5m from the stage. A halogen lamp was used for lighting (Arrilite 750 Plus ARRI, Munich, Germany). Constant angles of -50*^◦^* and 50*^◦^* were maintained between the axes of the halogen lamp and the axis of the hyperspectral camera. A certified reflectance standard (Labsphere, SRS-40-010) was placed next to the tray containing the hundred bays in order to know the reference reflected intensity (*I_o_*(*λ*)). This procedure allows to standardise the images coming from the non-uniformities of the instrumentation (light source, lens, detector). For each image, spectra of the berries were selected by using the Spectral Angle Mapper (SAM) and were averaged.

### 2.3 Data analysis

All the calculations were run under Matlab (The Mathworks, Natick, MA, USA). PLS-R algorithm was used to perform model sugar content [3]. Model results for each varieties were evaluated on the basis of the coefficient of determination (R^2^*_cv_*) and the root mean square standard error of cross-validation (RMSE*_cv_*).

## CRediT authorship contribution statement

**Maxime Ryckewaert:** Conceptualization, Investigation, Methodology, Software, Supervision, Writing – original draft. **Daphné Héran:** Methodology, Conceptualization, Writing – review & editing, Investigation. **Carole Feilhes:** Methodology, Conceptualization, Data curation, Visualization, Investigation, Resources. **Fanny Prezman:** Methodology, Conceptualization, Data curation, Resources. **Eric Serrano:** Supervision, Validation, Resources. **Aldrig Courand:** . **Silvia Mas-Garcia:** Methodology, Conceptualization. **Maxime Metz:** Methodology, Software, Resources. **Ryad Bendoula:** Investigation, Supervision, Writing – original draft, Resources.

## Declaration of Competing Interest

The authors declare that they have no known competing financial interests or personal relationships that could have appeared to influence the work reported in this paper.

## Data Availability

Spectral dataset of grape berries from hyperspectral imaging for maturity monitoring (Original data) (Mendeley Data)
